# A spatial analysis of variations in health access: linking geography, socio-economic status and access perceptions

**DOI:** 10.1186/1476-072X-10-44

**Published:** 2011-07-25

**Authors:** Alexis J Comber, Chris Brunsdon, Robert Radburn

**Affiliations:** 1 Department of Geography, University of Leicester, Leicester, LE1 7RH, UK; 2 Department of Geography, University of Liverpool, Liverpool, L69 7ZT, UK; 3 Research and Insight Team, Leicestershire County Council, Leicester, LE3 8RA, UK

**Keywords:** Accessibility, Geographically Weighted Regression

## Abstract

**Background:**

This paper analyses the relationship between public perceptions of access to general practitioners (GPs) surgeries and hospitals against health status, car ownership and geographic distance. In so doing it explores the different dimensions associated with facility access and accessibility.

**Methods:**

Data on difficulties experienced in accessing health services, respondent health status and car ownership were collected through an attitudes survey. Road distances to the nearest service were calculated for each respondent using a GIS. Difficulty was related to geographic distance, health status and car ownership using logistic generalized linear models. A Geographically Weighted Regression (GWR) was used to explore the spatial non-stationarity in the results.

**Results:**

Respondent long term illness, reported bad health and non-car ownership were found to be significant predictors of difficulty in accessing GPs and hospitals. Geographic distance was not a significant predictor of difficulty in accessing hospitals but was for GPs. GWR identified the spatial (local) variation in these global relationships indicating locations where the predictive strength of the independent variables was higher or lower than the global trend. The impacts of bad health and non-car ownership on the difficulties experienced in accessing health services varied spatially across the study area, whilst the impacts of geographic distance did not.

**Conclusions:**

Difficulty in accessing different health facilities was found to be significantly related to health status and car ownership, whilst the impact of geographic distance depends on the service in question. GWR showed how these relationships were varied across the study area. This study demonstrates that the notion of access is a multi-dimensional concept, whose composition varies with location, according to the facility being considered and the health and socio-economic status of the individual concerned.

## 1. Introduction

The subject of health facility access has long been of concern to community and public health planners [1-4]. Previous research on public health access has been in two distinct and usually non-overlapping areas. One tranche has considered the spatial dimensions related to geographic access (distances, travel times, catchments, etc), with data being manipulated and geographically analysed using geographical information systems (GIS) before subsequent statistical analyses [5-8]. Another body of research has examined service accessibility by considering the socio-economic aspects of access related to cost, insurance provision etc, with data collected using opinion or attitudes surveys [9-13]. In both cases the objective is usually to inform spatial planning and health policy making. This paper presents an analysis that straddles these different types of accessibility research. It uses a local regression analysis (as opposed to a global one) to explicitly link the experiential and geographical dimensions of access in order to provide a more nuanced and comprehensive analysis of health facility access. It combines analyses of public perceptions of service accessibility from an attitudes survey with an analysis of geographic road distance to those services. The attitudes survey captured information on the difficulty experienced by respondents in their access to different medical facilities, respondent health status and car ownership.

The primary aim of this study was to determine local spatial variations in the statistical relationships between perceptions of health facility access with geographical distance to the nearest facility, health status and car ownership. Examining the spatial non-stationarity in these relationships identifies locales where mismatches between access perceptions and geographic access exist, thereby allowing community health planners to target different activities in those specific areas. For example, areas where negative perceptions of access are not predicted by geographic distance and health status may be indicative of some underlying problem in service delivery. A secondary aim was to explore the different dimensions associated with the concept of 'accessibility' that ought to be considered in health planning. This was done by analysing access attitudes in combination with access geographies. Hitherto, much of the health geographics literature has only been concerned with physical or geographic distance. The use of local regression techniques to accommodate any spatial non-stationarity builds on and extends previous work that has considered the different dimensions associated with service access [14].

## 2. Background

The 'spatial' or geographic aspects of health provision and access to facilities have been considered in much previous research. Typically in such studies distances to services or facilities are measured (straight line or road distance) and analysed in order to quantify differences in access, gaps in service provision, to model optimal facility location and to identify inequalities in service provision. Recent examples of these purely spatial approaches in health science include identifying health catchments [5,15], examining equity of access for different social groups [8,16,17] and modelling spatial patterns of facility usage and access [18-21]. Additionally, a number of reviews of the use of GIS based technologies to evaluate geographic or physical access to health services have been published [22-25]. This body of research applies geographic and spatial statistics to determine how best to allocate resources in order to minimise gaps in provision and to identify service users with low levels of access. Increasing sophistication in analysis is also evident with evaluations of different distance measures relating to access [26,27], alternative statistical models [21], exploration of geographical variation in access models [27-29] and advanced heuristic search techniques for optimising facility locations [1,30]. However, whilst these various analyses have in some cases recognised the different dimensions of accessibility [31], they have generally adopted a specifically spatial or geographic definition of 'access',-i.e. one based on quantitative analyses of distances and travel times to services to define service accessibility.

The concepts of 'Access' and 'Accessibility' are more complex than simple distance measures [32,33]. They encompass a wider set of factors relating to behaviours and perceptions which relate to a range of highly qualitative factors such as perceived service quality, opening hours and previous experiences. From the social sciences literature Farrington and Farrington note that accessibility can be viewed as "the ability of people to reach and engage in opportunities and activities" [[34], p2] and therefore improving access outcomes involves overcoming the social dimensions of access and separation, as well as spatial constraints. Multi-dimensional approaches in health planning have been recommended, ones that consider aspects other than distance and cost, in order to identify different barriers to health care services [35]. However, in only a few cases where qualitative and quantitative access dimensions have been considered, were the local spatial variations in the relationships examined. For example, Maroko et al [33] used Geographically Weighted Regression to explore the spatial relationships between the variables associated with models of park acreage and density of physical activity sites.

There has been little research that has explicitly examined the spatial variation of factors related to access perceptions against geographic factors. The purpose of this research was to address such gaps. First, it examined how the perceptions of access to health facilities, as captured by an attitudes survey, related to geographical or spatial measures of access and health status globally, using a generalized linear model (GLM). Second, the spatial variations in these relationships were analysed using Geographically Weighted Regression (GWR), a local regression analysis which allows the spatial non-stationarity of relationships between variables to be examined. The models resulting from these two approaches were used to predict respondent perceptions over service access from stated health status, network distance to the nearest facility and car ownership. By analysing access perceptions and access distances to hospitals and GPs this research also compares how these relationships vary for different types of health service.

## 3. Methods

### Data and Study Area

**Figure 1:**
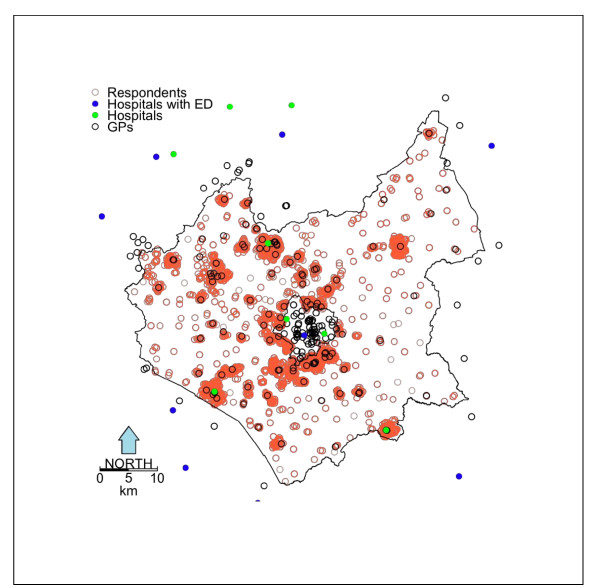
**The study area, Leicestershire UK, and the locations of the attitude survey respondent postcodes, GP surgeries, hospitals and hospitals with Emergency Departments (ED)**.

An attitudes survey in the UK county of Leicestershire was conducted in 2008 by Leicestershire County Council (LCC) as part of the UK government's Department of Communities and Local Government's 'Place Survey'. Leicestershire is a rural county, with the City of Leicester (a separate local authority) forming a hole in centre of the county (see Figure 1). The Place Survey is a postal survey designed to collect data to support national indicators. Individual local government authorities were responsible for administering the survey and were able to include additional questions if they so wished. Because of this, LCC included questions that asked respondents to describe their perceptions of their access to a range of health services (GP surgeries, dentists, hospitals and pharmacies) using a 5-point scale that allowed respondents to indicate whether they found access 'Very easy', 'Fairly easy'. 'Neither easy nor difficult', 'Fairly difficult' or 'Very difficult'. Respondents were also asked to indicate their general health (a 5-point scale from very good to very bad), whether they had any long-standing illness, disability or infirmity (yes or no) and whether they owned a car or not. In Leicestershire there were 8530 responses to the Place Survey, with 4.9% indicating difficulty (i.e. replying either 'difficult' or 'very difficult') in their access to GPs and 20.2% indicating difficulty in their access to hospitals. Of the respondents, 4.6% stated that they had 'bad health' or 'very bad health' (henceforth 'Bad Health'), 33.1% indicated that they had some Long Term Illness and 16.0% stated that they did not own a car (henceforth 'Non-Car Ownership'). The sampling frame for the Place Survey selected household addresses at random from the Post Office small users Address File database. For each of the 7 districts in Leicestershire, sampling was stratified with the aim of reaching a sample size of at least 1,100 in each district, regardless of population size. Central government provided the sample of addresses. The questionnaire was sent to households only and was completed by any resident aged 18 or over living at the address. A total of 20,260 questionnaires were sent out and the response rate for each district was between 41% and 43%. The survey response rates by demographic factors are summarised in Table 1. Leicestershire Statistics and Research Online provide detail of the Place Survey in Leicestershire^1 ^and an interactive visualisation of the results^2^.

**Table 1:** The summary of the Place Survey response rates.

	**Factors**									
	
	**Age**	***Count***	**Health**	***Count***	**Disability**	***Count***	**Gender**	***Count***	**Ethnicity**	***Count***
	
	18 to 24	145	Very good	2377	Limiting	1913	Female	4816	White British	7949
	25 to 44	1839	Good	3622	Non-Limiting	911	Male	3530	BME	416
	45 to 64	3187	Fair	1958	None	5425				
	65 to 74	1561	Bad	333						
	75 to 84	1104	Very bad	60						
	85 +	348								
										
**Not provided**		346		180		281		184		165
	
**Totals**		8530		8530		8530		8530		8365

BME is 'black and minority ethnicity'.

In the UK GP surgeries provide free access to a medical practitioner who treats acute and chronic illnesses, provides preventive care and health education for all. Data for GP surgeries and major National Health Service (NHS) hospitals, with and without Emergency Department (ED) facilities, were downloaded from the NHS website http://www.nhs.uk and spatially located from their postcodes. In the UK there are an average of ~15 residential addresses per postcode providing a fine level of geographical detail. The locations of GP surgeries, hospitals and Place Survey respondents are shown in Figure 1. The road data was the Ordnance Survey MasterMap Integrated Transport Network layer provided via the EDINA data library http://edina.ac.uk/. A GIS-based network analysis (ArcGIS 9.3) was used to calculate the road distance from each Place Survey post-code location respondent to the nearest GP surgery, hospital and hospital with ED facilities. All of the statistical analyses and mappings were performed in R version 2.13.0, the open source statistical software http://cran.r-project.org/.

### Analysis

The Place Survey data were analysed using Generalized Linear Models (GLMs), which predict the response coefficients from a linear predictor generated from the independent terms. A logistic GLM was used to analyse the extent to which different variables predict difficulty in access to GPs and Hospitals. The *logit *function is defined by(1)

The dependent variable was the survey response to the appropriate access question. A value of 1 was given for a response of 'difficult' or 'very difficult', a value of 0 for any other response. The first independent variable tested was whether the respondent had Long Term Illness. The GLM was then extended to include respondent statements on their health status (Bad Health), car ownership and their distance to the nearest facility as measured using a GIS-based network analysis.

For ease of access to GPs, three models were considered:(2)(3)(4)

and(5)

where y_1 _is a 0/1 indicator showing whether the respondent expressed difficulty in their access to GPs, x_1 _is an 0/1 indicator variable showing whether the respondent said they had a Long Term Illness, x_2 _is an indicator variable stating whether the respondent considered they were in Bad Health, x_3 _is the distance from the respondent to their nearest GP surgery based on road network distance and x_4 _is a 0/1 indication of car ownership.

The quantity *exp*(*b_i_*) gives the odds ratio associated with a unit increase in x_i _- that is the ratio between the odds of a y-value of 1 for x_i _and a y-value of 1 if x_i _is replaced by x_i _+ 1. These values, together with 95% confidence intervals, are given in Table 2. All three models were compared using Akaike's Information Criterion (AIC).

**Table 2:** Results of the GLM analyses of dissatisfaction over access to doctors/GP (Models 1 to 4) and hospitals (Models 5 to 9)-for each set of models, the best AIC value is highlighted in **bold**.

Analysis	Model	Variable	Odds ratio	Lower 95% CI	Upper 95% CI	AIC
Access to GP surgeries	Model 1	Long Term Illness	2.27	1.86	2.76	3257.9
	
	Model 2	Long Term Illness	2.00	1.62	2.46	3242.9
			
		Bad Health	2.10	1.49	2.90	
	
	Model 3	Long Term Illness	2.07	1.68	2.56	3181.1
			
		Bad Health	2.10	1.49	2.92	
			
		Geographic Distance (to nearest GP)	1.29	1.22	1.36	
	
	Model 4	Long Term Illness	1.80	1.45	2.24	**3049.5**
			
		Bad Health	1.69	1.18	2.37	
			
		Geographic Distance (to nearest GP)	1.34	1.27	1.42	
			
		Non-Car Ownership	3.81	3.06	4.72	

Access to Hospitals	Model 5	Long Term Illness	1.42	1.27	1.58	8549.2
	
	Model 6	Long Term Illness	1.32	1.18	1.48	8535.5
			
		Bad Health	1.61	1.28	2.02	
	
	Model 7	Long Term Illness	1.32	1.18	1.48	8537.5
			
		Bad Health	1.61	1.28	2.02	
			
		Geographic Distance (to nearest Hospital)**	1.00	0.99	1.01	
	
	Model 8	Long Term Illness	1.32	1.18	1.48	8532.7
			
		Bad Health	1.61	1.28	2.03	
			
		Geographic Distance (to nearest ED Hospital)*	0.991	0.982	0.999	
	
	Model 9	Long Term Illness	1.26	1.12	1.42	**8488.4**
			
		Bad Health	1.50	1.19	1.89	
			
		Geographic Distance (to nearest ED Hospital)*	0.991	0.982	0.999	
			
		Non-Car Ownership	1.61	1.41	1.84	

All variables significant at the 1% level except where indicated at 5% (*) and as not significant (**).

For access to hospitals, similar models were fitted, as below.(6)(7)(8)(9)

and(10)

where x_1_, x_2 _and x_4 _are as above, y_2 _is a 0/1 indicator variable showing whether the respondent stated that they experienced difficulty in their access to hospitals, x_3 _is the distance from the nearest hospital to the respondent's address, and x_3a _is the distance from the nearest hospital *with an Emergency Department *to the respondent's address. As before, coefficients were estimated, odds ratios computed and different models were compared using AICs.

### Geographic Variation

The use of linear regression is common in many areas of science. Ordinary linear regression implicitly assumes spatial stationarity of the regression model-that is, the relationships between the variables remain constant over geographical space. It is self evident that global averages of spatial data are not always helpful, whether they are related to health, or other domains (e.g. unemployment or climate). Spatial non-stationarity occurs when a relationship (or pattern) that applies in one region does not apply in another. Global models are statements about processes or patterns which are assumed to be stationary and as such are location independent-i.e. are assumed to apply in all locations. In contrast, local models are spatial disaggregations of global models, the results of which are location-specific. The template of the model is the same: the model is a linear regression model with certain variables, but the coefficients alter geographically. The above is essentially a description of Geographically Weighted Regression [36-38] (GWR). One of the fundamental tenets of geographical analyses is to evaluate the potential existence of spatial variability of statistical models. GWR allows one to consider and test for the possibility that relationships vary geographically. It is an approach that deals with spatial non-stationarity in multivariate regression by estimating regression coefficients locally using spatially dependent weights, under the assumption that the effect of the predictor variables on the dependent variable will vary continuously over space. The logistic regressions of Model 4 and Model 9 were extended to a GWR analysis as follows:(11)(12)

with the coefficients for each of the predictor variables assumed to vary across the two-dimensional geographical space defined by the coordinates (*u*, *v*). Consequently the coefficients in GWR can be considered as functions of these coordinates, rather than single-valued variables.

## 4. Results

The results of applying the GLM are shown in Table 2, with Models 1 to 4 relating to access to GPs and Models 5 to 9 relating to hospital access.

### Access to GPs

Model 1 shows that Long Term Illness is a significant predictor of experiencing difficulty in access to GPs. The inclusion of additional terms (Models 2, 3, 4) each improved the model as shown by the decreasing AIC score. AIC is minus twice the maximized log-likelihood plus twice the number of parameters, as computed by the AIC component of the family. For the binomial family of models, the dispersion is fixed at one and the number of parameters is the number of coefficients. The inclusion of health status (Model 2), distance to the nearest GP surgery (Model 3) and Non-Car Ownership (Model 4) significantly improved the model. Non-Car Ownership was significant at the 99% level and the AIC decreased between Models 3 and 4 by around 132 points. The analysis of deviance tests between Model 1, Model 2, Model 3 and Model 4 confirm the significance of these variables (Table 3).

**Table 3:** Analysis of Deviance of the terms associated with dissatisfaction over access to doctors, *** indicates significance at the 0.1% level, * indicates significance at the 5% level.

Analysis	Terms	Df	Residual Df	Residual Deviance	Deviance Reduction
Access to GP surgeries	NULL		8529	3318.6	
	
	Long term Illness	1	8528	3253.9	64.764***
	
	Bad Health	1	8527	3236.9	16.975***
	
	Distance to nearest GP surgery	1	8526	3173.1	63.813***
	
	Non-Car Ownership	1	8525	3039.5	133.56***

Access to Hospitals	NULL		8529	8583.8	
	
	Long term Illness	1	8528	8545.2	38.611***
	
	Bad Health	1	8527	8529.5	15.655***
	
	Distance to the nearest ED Hospital	1	8526	8524.7	4.826*
	
	Non-Car Ownership	1	8525	8478.4	46.295***

Analysis of the exponentials of the coefficient estimates (Table 2) allows the odds ratios and confidence intervals associated with different factors to be calculated. The odds ratios calculated from Model 4 coefficients suggest the following statements:

- For respondents with Long Term Illness the relative odds of experiencing difficulty in access to GPs are around 1.8 times greater than for those who not have Long Term Illness;

- For respondents with Bad Health the relative odds of experiencing difficulty are around 1.7 times greater than for those who not have Bad Health;

- The relative odds of experiencing difficulty in access to GP surgeries increases by 34% per extra km to the nearest GP surgery;

- Non-Car Ownership was found to have a profound impact on GP access perceptions. For those who not own a car, the relative odds of experiencing difficulty over access to GPs are 3.8 times more than for those who do own cars.

### Access to hospitals

Model 5 shows Long Term Illness to be a significant predictor of experiencing difficulty in access to hospitals. The model was extended to include the additional terms of Bad Health, distance to the nearest hospital and to the nearest Emergency Department facility, as measured using a GIS-based network analysis, and car ownership. The inclusion of the health terms (Model 6) improved the model, but distances to the nearest hospital did not (Model 7). Distance to the nearest hospital with an ED improved the model slightly (Model 6 to Model 8)-ED hospital distance was significant only at the 95% level-whilst Non-Car Ownership (Model 9) again significantly improved the model. In this case the AIC decreased by around 44 points between Models 8 and 9. The results of analysis of deviance tests between Model 5, Model 6, Model 8 and Model 9 confirm the significance of these variables (Table 3).

The odds ratios associated with different factors and models in relation to difficulty in accessing hospitals are shown in Table 2. The odds ratios calculated from the Model 9 coefficients suggest the following statements:

- For respondents with Long Term Illness the relative odds of experiencing difficulty in access to hospitals are around 26% greater than for those who not have Long Term Illness;

- For respondents with Bad Health the relative odds of experiencing difficulty are around 50% greater than for those who not have Bad Health;

- Whilst distance to hospitals was not found to be a good predictor of difficulty in hospital access, distance to hospitals with EDs was significant but negative. The relative odds decreased slightly (1%) with each extra km distance to the nearest ED hospital;

- The impact of Non-Car Ownership was again profound: for those who do not own a car the relative odds of experiencing difficulty over access to hospitals are 61% greater than for those who do own cars.

### Geographic Variation

To complement the logistic regression above and to examine the spatial variation in these relationships, GWR was used to generate spatially explicit logistic regression models. Table 4 summarises the results of the two GWR analyses (Equations 11 and 12) and describes the variation of the odds ratios for the different independent variables. The Inter-Quartile Range of the odds ratios provides a good indicator of the spatial variation. For Access to GPs, there was little spatial variation in Distance and Long Term Illness as predictors of access difficulty, whilst Bad Health showed some variation, with the relative odds of experiencing difficulty in access to this service ranging from 69% to 81% greater than for who do not have Bad Health. There was more variation in the effects of Non-Car Ownership, which ranged from 3.58 to 3.94 times greater than for those with cars, although the 25^th ^percentile is close to the median, indicating a positive skew in the distribution of the variation. For access to ED hospitals the relative odds of experiencing difficulty with Bad Health ranged from 35% to 64% greater than for those without Bad Health. The effects of Non-Car Ownership were greater but with similar spatial variation, and the relative odds of experiencing difficulty ranged from 47% to 73% greater than for those who owned a car.

**Table 4:** The variation in the odds ratios of the independent variables from the GWR models of access dissatisfaction, with the Inter-Quartile Range (IQR) providing a measure of the spatial variation the relationships.

GWR model	Variable	Minimum	1^st ^Quartile	Median	3^rd ^Quartile	Maximum	Global	IQR
GPs	Distance to nearest GP	1.32	1.33	1.34	1.37	1.41	1.34	0.04

	Bad Health	1.61	1.63	1.68	1.77	1.90	1.69	0.14

	Long Term Illness	1.69	1.77	1.80	1.81	1.82	1.80	0.04

	Non-Car Ownership	3.41	3.58	3.64	3.94	4.26	3.81	0.36

ED Hospital	Distance to nearest Hospital	0.798	0.981	0.987	0.992	1.240	0.991	0.011

	Bad Health	0.553	1.35	1.55	1.64	3.92	1.50	0.29

	Long Term Illness	0.800	1.21	1.24	1.27	1.99	1.26	0.06

	Non-Car Ownership	1.08	1.47	1.55	1.73	3.50	1.61	0.26

**Figure 2:**
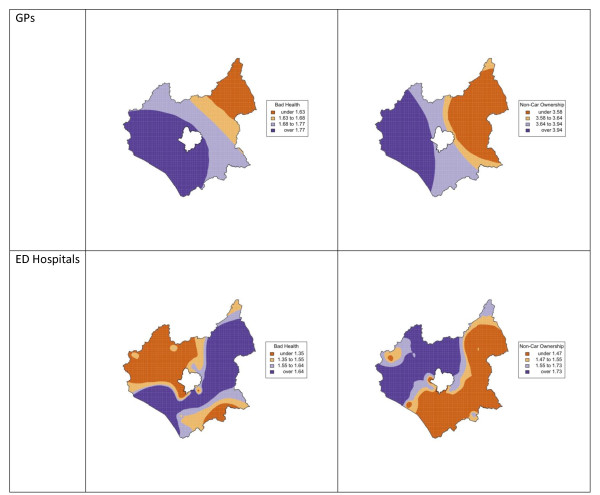
**Spatial variation in the relationships between perceived difficulty in access to hospitals and GPs with Bad Health and Non-Car Ownership**.

The spatial distribution of the relationships between access difficulty and the predictor variables showing high variation can be mapped. Figure 2 shows the spatial variation in the predictive strength of Bad Health and Non-Car Ownership on perceptions of access to GPs and Hospitals with ED. Table 3 shows that the other variables, whilst significant, did not vary spatially-i.e. the global model for these variables can be assumed to be unaffected by spatial non-stationarity. For access to GPs there is a clear trend of increasing perceived difficulty in access for those with Bad Health and who do not own cars running from the North East to the South West. For access to Hospitals, the spatial variations in the relationship with Bad Health on and Non-Car Ownership are not so even. There is much more short-range variation in the trends and clusters are evident in different parts of the study area, in contrasts to the general trend observed in GP access difficulty. The impact of Bad Health is greatest in a band running to the South and East of the study area and lowest in the North West and South East. The impact of Non-Car Ownership is greatest in the North West and least in a band running from the South and East.

## 5. Discussion

In this study area, perceptions of difficulty in access to different types of health services (hospitals and GPs) was found to be significantly related to Long Term Illness, Bad Health and Non-Car Ownership. Geographic distance was a significant predictor of perceived difficulty in access to GP surgeries but not for hospitals with or without EDs. A GWR analysis identified considerable geographic variation in the relationships between perceived difficulty in access to GPs and hospitals with Bad Health and Non-Car Ownership but not with Long Term Illness or geographic distance. For instance, difficulty in accessing GP surgeries in relation to Bad Health was greater in the West and South West of the study area. Whilst difficulty in accessing hospitals in relation to Bad Health was greater in the South West and North East.

These results reflect the variation in perceived ease of access to services within and between different groups defined on health status, socio-economic attributes, distance etc and that different factors are correlated with access difficulties, depending on the service. They suggests the following statements for this study area:

1) Distance is a significant factor in perceived difficulties in access to GPs but with little local variation. The notion of GP accessibility is strongly related to geographic distance.

2) Distance is not a factor in perceptions of hospital accessibility.

3) Long Term Illness and Bad Health are significant predictors of perceived difficulties in accessing GPs and hospitals, indicating that for people in these groups, the notion of accessibility is also related to their health status.

4) Non-Car Ownership was found to be a significant predictor of perceived difficulties in accessing GPs and hospitals indicating that the notion of accessibility is also related to the choices afforded by socio-economic status.

5) Additionally, the impacts of health status (Bad Health) and socio-economic status (Non-Car Ownership) on the perceived difficulties in access services were found to vary spatially, suggesting that other local factors may also be contributing to perceptions and notions of accessibility.

The results highlight some important issues related to service accessibility for consideration in health planning research. These relate to the *choices *available to and made by individuals over the services they use: perceptions of access will be influenced by their personal (health) circumstances and experiences. Whilst distance has been found to an important and significant determinant in health outcomes [39,40], patient access to and use of services will depend on a number of interacting factors, including socio-economic ones [41] and health status. For these reasons, other research has sought to combine a range of different measures in order to generate access indices that incorporate potential socio-economic and health barriers to services as well as geographic factors [41,42]. The perception of access to any given facility or service will also be related to other factors, and the choices made by individuals over which services to access (if they exist) will reflect these: cost, previous experience, reputation (first and second hand), perceived quality of service, convenience etc. The extent to which we have active choices in the services we access will also vary depending on the service in question: there may be little choice over the hospital ED we use, a bit more choice over which specialist hospital clinic we are (or choose to be) referred to and yet further choice over which GP we visit. These levels of choice are reflected in the results of this work: distance was significant factor in respondent perceptions over their access to GPs and not to hospitals. For these reasons, Place Survey respondents with ongoing health problems may be more likely to be concerned about access to their regular outpatient or inpatient treatment centres than to the emergency department. The variable impact of distance over perceptions of access highlights an important point: the concepts of facility 'access' and 'accessibility' involve much more than just geographic or spatial access [32,33]. Much spatial planning in public health and other domains is predicated on the assumption that geographic distance is important *per se *regardless of the nature of the facility, whereas this research has shown that this may not be the case. Notions of 'Access' or 'Accessibility' in a health planning context has been shown to involve considerations of:

*Financial access *relating to measures that describe the financial ability of people to access health care through health insurance and other cost-related barriers [43,44];

*Behavioural access*, describing actual utilization of different health services such as visits, prescription uptake, ambulatory calls [45-47];

*Spatial access *relating to geographic distance, transport and travel times and describing service catchments, optimal spatial arrangement of resources [1,5,8,30,48].

Whilst some public health studies incorporate multiple definitions of access [32,33,47], in general the literature describes access as relating to the cost barriers associated with health [35]. For example, guidelines produced by the National Academy of Sciences include access as a quality criterion and as an objective of health practice but adopt a cost based concept of access related to insurance [35]. In other research domains notions of access relate to social justice, social inclusion, environmental justice, public participation and public engagement. For example, in the health GIS literature access is described in relation to geographic distance [1,5,15,22-25,48], whilst in the social sciences it is related to access perceptions [34] and notions of social capital [49-51]. This study has highlighted the need for research on access to public health facilities to accommodate the different dimensions of access, that relate to geography, behaviours and perceptions as well as financial and cultural barriers.

Some limitations to this study should be noted. The hospital data was downloaded from the NHS website to include 'major' NHS Hospitals. However, survey respondents were simply asked about their perceptions of access to 'hospitals' which, depending on their personal experiences may include children's hospitals and long stay psychiatric facilities. The attitudes survey captured the degree of difficulty experienced in accessing services but not the underlying reasons for that difficulty. Similarly, the analysis uses geographic distance to the nearest facility, which may or may not be the facility actually used by the survey respondents. However, the responses do provide an indication of the exclusion experienced by a robust sample of the population in the study area. Ongoing work will seek to unpick the underlying causes of the negative perceptions of access. The data used in the study did not capture any information about use and access to *private *facilities, such as are available under personal health insurance schemes.

This analysis did not account for age and gender for a number of reasons. First, the main messages from this research are that local statistical techniques can add considerable value to accessibility (and other) analyses by identifying spatial variation in relationships, and that access perceptions are driven by different factors (distance, health status, etc) depending on the facility under consideration. Second, whilst in epidemiological studies age and gender are included to adjust for the 'population at risk', with certain kinds of illness more likely to occur in age groups than in others, this work does not assess the relative occurrences of diseases over geographical space but changes in perceptions of accessibility. From a policy viewpoint it is useful to consider this for populations as a whole, even if the composition of such populations varies geographically. Third, a larger sample would be required to calibrate the GWR model reliably to analyse population subsets and to allow for the geographical factor. Therefore a pooled analysis was carried out because of the geographical detail required and the danger of small sample problems. It may be appropriate at a later stage to consider the factors of age and gender in the light of the findings outlined here.

## 6. Conclusions

This study demonstrates that the notion of access is a multi-dimensional concept, whose composition varies with location, according to the facility being considered and the health and socio-economic status of the individual concerned. Some conclusions from this study (and this study area) can be drawn:

• For some types of health facilities geographic distance is a significant predictor of experiencing access difficulty (GPs), whilst for others it is not (hospitals);

• Those with Long Term Illness and Bad Health status are much more likely to experience difficulty in their access to health facilities;

• Non Car Ownership was found to be significantly related to access difficulty;

• Some of these relationships vary spatially indicating the need for accessibility analyses to include spatially nuanced statistical methods that accommodate local variations, such as are afforded by GWR.

• Identifying the spatial variations in relationships, by estimating local regression parameters, allows the spatial distribution and interaction of predictor variables to be explored. Analysing the local variation in relationships provides those concerned with public health policy with the ability to target resources and to achieve improved outcomes through location-specific activities.

• Future analyses of facility access and accessibility should seek to include the different dimensions related to service access including: public perceptions, behaviours, geographical access and service quality. These were found to provide a more comprehensive analysis of health service access when considered together.

## Competing interests

The authors declare that they have no competing interests.

## Authors' contributions

AC conceived designed and implemented the analysis including exploratory data analysis and visualization of data and data processing. RR developed the accessibility components to the questionnaire and initiated this investigation. AC led the writing. CB supervised the statistical analysis. All of the authors have read and approved the final manuscript and contributed to the writing.

## Endnotes

^1^http://www.lsr-online.org/leicestershire-place-survey-2008.html

^2 ^http://www.lsr-online.org/placesurvey.html
